# The semi-automation of title and abstract screening: a retrospective exploration of ways to leverage Abstrackr’s relevance predictions in systematic and rapid reviews

**DOI:** 10.1186/s12874-020-01031-w

**Published:** 2020-06-03

**Authors:** Allison Gates, Michelle Gates, Meghan Sebastianski, Samantha Guitard, Sarah A. Elliott, Lisa Hartling

**Affiliations:** 1 Alberta Research Centre for Health Evidence, Department of Pediatrics, University of Alberta, Edmonton, Alberta Canada; 2 Alberta Strategy for Patient-Oriented Research (SPOR) SUPPORT Unit Knowledge Translation Platform, University of Alberta, Edmonton, Alberta Canada

**Keywords:** Systematic reviews, Rapid reviews, Machine learning, Automation, Efficiency

## Abstract

**Background:**

We investigated the feasibility of using a machine learning tool’s relevance predictions to expedite title and abstract screening.

**Methods:**

We subjected 11 systematic reviews and six rapid reviews to four retrospective screening simulations (automated and semi-automated approaches to single-reviewer and dual independent screening) in Abstrackr, a freely-available machine learning software. We calculated the proportion missed, workload savings, and time savings compared to single-reviewer and dual independent screening by human reviewers. We performed cited reference searches to determine if missed studies would be identified via reference list scanning.

**Results:**

For systematic reviews, the semi-automated, dual independent screening approach provided the best balance of time savings (median (range) 20 (3–82) hours) and reliability (median (range) proportion missed records, 1 (0–14)%). The cited references search identified 59% (*n* = 10/17) of the records missed. For the rapid reviews, the fully and semi-automated approaches saved time (median (range) 9 (2–18) hours and 3 (1–10) hours, respectively), but less so than for the systematic reviews. The median (range) proportion missed records for both approaches was 6 (0–22)%.

**Conclusion:**

Using Abstrackr to assist one of two reviewers in systematic reviews saves time with little risk of missing relevant records. Many missed records would be identified via other means.

## Background

Systematic evidence syntheses provide the foundation of informed decision-making; however, the large and growing body of primary studies makes it difficult to complete them efficiently and keep them up-to-date [1]. To avoid missing relevant studies, rigorously conducted evidence syntheses typically include comprehensive searches of multiple sources [2]. Often, two reviewers screen through the records retrieved, first by title and abstract and then by full text, to identify those that are relevant. The process requires substantial effort and time to return a relatively small body of relevant studies [1]. Machine learning (ML) tools provide the potential to expedite title and abstract screening by predicting and prioritizing the relevance of candidate records.

At the time of writing the SR Tool Box, an online repository of software tools that support and/or expedite evidence synthesis processes, referenced 37 tools aimed at supporting title and abstract screening [3]. Freely-available, off-the-shelf tools like Abstrackr, RobotAnalyst, and Rayyan allow review teams without ML expertise and/or limited resources to create efficiencies during title and abstract screening. By prioritizing relevant records, such tools provide reviewers with the opportunity to identify relevant studies earlier and move forward with subsequent review tasks (e.g., data extraction, risk of bias appraisal) sooner [4]. The relevance predictions produced by ML tools can also be leveraged by review teams to semi-automate title and abstract screening by eliminating records predicted to be irrelevant [5].

Mounting interest in the use of ML tools to expedite title and abstract screening has been accompanied by skepticism and distrust by review teams and end users of reviews, and adoption has been slow [6]. A fundamental concern associated with automatically or semi-automatically eliminating candidate records is that important studies may be missed, compromising the comprehensiveness of the review and potentially the validity of its conclusions. Evidence of reliable ways to leverage ML tools’ relevance predictions in real-world evidence synthesis projects is one step toward garnering trust and promoting adoption. In the present study, our objective was to explore the benefits (workload and estimated time savings) and risks (proportion of studies missed) of leveraging a ML tool’s predictions to expedite citation screening via four retrospective screening simulations.

## Methods

### Protocol

In advance of the study the research team developed a protocol, available via the Open Science Framework (https://osf.io/2ph78/, doi: 10.17605/OSF.IO/2PH78). We undertook the following changes to the protocol during the conduct of the study: (1) added an additional systematic review to the sample; and (2) added a post-hoc analysis to determine if missed studies would have been located by scanning reference lists. We added the additional systematic review prior to data analysis, as it had recently been completed at our centre and allowed for a larger sample of reviews. The post-hoc analysis was added recognizing that electronic database searching is just one of the means by which relevant studies are typically sought in systematic reviews.

### Abstrackr

Abstrackr is a freely available ML tool (http://abstrackr.cebm.brown.edu) that aims to enhance the efficiency of title and abstract screening [7]. To screen in Abstrackr, all citations retrieved via the electronic searches must first be uploaded to the software. The reviewer is then prompted to select review settings, including how many reviewers will screen each title and abstract (one or two), and the order in which the records will be viewed (in random order, or by predicted relevance). Once the review is set up, records are presented to reviewers one at a time on the user interface, including the title, authors, abstract, and keywords. As records appear on the screen, the reviewer is prompted to label each as relevant, irrelevant, or borderline, after which the next record appears.

While reviewers screen in Abstrackr, the ML model learns to predict the relevance of the remaining (unscreened) records via active learning and dual supervision [7]. In active learning, the reviewer(s) must first screen a “training set” to teach the model to distinguish between relevant and irrelevant records based on common features (e.g., words or combinations or words that are indicative of relevance or irrelevance). In dual supervision, the reviewers can impart their knowledge of the review task to the model in the form of labeled terms. When setting up the review, reviewers can tag terms that are indicative of relevance or irrelevance. For example, the terms “systematic review” or “review” may be tagged as irrelevant in systematic reviews that seek to include only primary research. The relevance terms are exploited by the model, along with the reviewers’ screening decisions, when developing predictions [7].

After screening a training set, the reviewers can view and download Abstrackr’s relevance predictions for the records that have not yet been screened. The predictions are typically available within 24 h of screening an adequate training set (i.e., upon server reload). The predictions are presented to reviewers in two ways: a numeric value representing the probability of relevance (0 to 1), and a binary relevance prediction (i.e., the “hard” screening prediction, true or false). Review teams may choose to leverage these predictions to prioritize relevant records, or to automatically eliminate records that are less likely to be relevant.

Although many ML tools aimed at expediting title and abstract screening exist, we chose Abstrackr for this study because: (1) its development is well documented; (2) empirical evaluations of its performance exist [8–11]; (3) experiences at our centre showed that it was more reliable and user-friendly than other available tools [11]; and (4) it is freely available, so more review teams are likely to benefit from practical evaluations of its capabilities.

### Sample of reviews

We selected a convenient sample of 11 systematic reviews and 6 rapid reviews completed at our centre. The reviews were heterogeneous with respect to the type of research question, included study designs, screening workload, and search precision (Table 1).

**Table 1:** Characteristics of the included reviews ^a^

Review name	Review question	Eligible study designs	Screening workload, n ^b^	Included, n (% of total)	
				Title and abstract	Full text
**Systematic Reviews**					
**Biomarkers**	Diagnostic accuracy	Any	1812	209 (12)	45 (2)
**Brain injury**	Diagnostic accuracy	RCTs, cohorts, case-control	6262	518 (8)	40 (1)
**Activity and pregnancy**	Exposure	Any	2928	236 (8)	98 (3)
**Concussion**	Exposure	Cross-sectional, cohorts, mixed methods, qualitative	1439	46 (3)	5 (< 1)
**Antipsychotics**	Intervention	RCTs, nRCTs, controlled cohorts, controlled before-after	12,156	1177 (10)	127 (1)
**Digital technologies for pain**	Intervention	RCTs, nRCTs, observational	2662	207 (8)	64 (2)
**Treatments for bronchiolitis**	Intervention	RCTs	5861	518 (9)	137 (2)
**VBAC**	Intervention	RCTs, nRCTs, controlled observational	5092	807 (16)	21 (< 1)
**Visual acuity**	Intervention	RCTs	11,229	224 (2)	1 (< 1)
**Experience of bronchiolitis**	Qualitative/mixed methods	Observational, qualitative, mixed methods	651	88 (14)	28 (4)
**Experiences of UTIs**	Qualitative/mixed methods	Observational, qualitative, mixed methods	1493	25 (2)	4 (< 1)
**Rapid reviews**					
**Preterm delivery**	Diagnostic accuracy	Systematic reviews, cohorts	451	96 (21)	34 (8)
**Community gardening**	Intervention	Any	1536	153 (10)	32 (2)
**Depression safety**	Intervention	RCTs, systematic reviews	964	44 (5)	8 (1)
**Depression treatments**	Intervention	Systematic reviews	1583	418 (26)	179 (11)
**Patient education for cancer**	Intervention	RCTs	2413	153 (6)	1 (< 1)
**Workplace stress**	Intervention	Systematic reviews	767	141 (18)	59 (8)

*nRCTs* non-randomized controlled trials; *RCTs* randomized controlled trials; *UTI* urinary tract infection; *VBAC* vaginal birth after caesarean section

^a^Sorted by review question, then alphabetically by review name

^b^Retrospective screening workload for each of the two reviewers in systematic reviews, and for the single reviewer in rapid reviews

The median (range) screening workload for the systematic reviews was 2928 (651 to 12,156) records. Across systematic reviews, 8 (2 to 16)% of the records retrieved by the searches were included at the title and abstract screening stage, and 1 (0.01 to 3)% following scrutiny by full text. The median (range) number of included records was 40 (1 to 137). The median (range) screening workload for the rapid reviews was 1250 (451 to 2413) records. Across rapid reviews, 14 (5 to 26)% of the records retrieved by the searches were included at the title and abstract screening stage, and 5 (0.04 to 8)% following scrutiny by full text. The median (range) number included records was 33 (1 to 179).

Although there can be several differences in conduct between systematic and rapid reviews, for the purpose of this study we defined the review types based solely on the method of study selection. For the systematic reviews, two reviewers independently screened all records at the title and abstract stage, and any record marked as relevant by either reviewer was scrutinized by full text. The two reviewers agreed on the full texts included in each review, and all disagreements were resolved through discussion or the involvement of a third reviewer. In all cases, the two reviewers included: (a) a senior reviewer (typically the researcher involved in planning and overseeing the conduct of the review, and the reviewer with the most systematic review and/or content experience), and (b) one or more junior reviewers (i.e., second reviewers), who were typically research assistants involved in screening and sometimes (but not always) other aspects of the systematic review (e.g., data extraction, risk of bias appraisal).

For the rapid reviews, a single, experienced (i.e., senior) reviewer selected the relevant studies, both at the title and abstract and full text stages. Compared with dual independent screening, the risk for missing relevant studies is increased when study selection is performed by a single reviewer; however, the approach is likely appropriate for rapid reviews [12]. We selected this approach in order to create efficiencies while maintaining an acceptable level of methodological rigour, in consultation with the commissioners and/or end users of each review.

### Screening procedure

For each review, we uploaded the records identified via the electronic database searches to Abstrackr and selected the single screener mode and random citation order setting. Abstrackr’s ability to learn and accurately predict the relevance of candidate records depends on the correct identification and labeling of relevant and irrelevant records in the training set. Thus, members of the research team (AG, MG, MS, SG) retrospectively replicated the senior reviewer’s (i.e., the reviewer we presumed would have made the most accurate screening decisions) original screening decisions based on the screening records maintained for each review, for a 200-record training set. Although the ideal training set size is not known, similar tools suggest a training set containing at least 40 excluded and 10 included records, up to a maximum of 300 records [13].

For systematic reviews conducted at our centre, any record marked as “include” or “unsure” by either of two independent reviewers is eligible for scrutiny by full text (i.e., the responses are deemed equivalent). Thus, our screening records include one of two decisions per record: include/unsure or exclude. It was impossible to retrospectively determine whether the “include/unsure” decisions were truly includes or unsures, so we considered all to be includes.

After screening the training sets, we waited for Abstrackr’s relevance predictions. When predictions were not available within 48 h, we continued to screen in batches of 100 records until they were. Once available, we downloaded the predictions. We used the “hard” screening predictions (true or false, i.e., relevant or irrelevant) rather than deciding on custom eligibility thresholds based on Abstrackr’s relevance probabilities. As the ideal threshold is not known, using the hard screening predictions likely better approximated real-world use of the tool.

### Retrospective simulations

We tested four ways to leverage Abstrackr’s predictions to expedite screening:

1. In the context of single reviewer screening (often used in rapid reviews):

- **Fully automated, single screener approach**: after screening a training set of 200 records, the senior reviewer downloads the predictions, excludes all records predicted to be irrelevant, and moves the records predicted to be relevant forward to full text screening; or
- **Semi-automated, single screener approach**: after screening a training set of 200 records, the senior reviewer downloads the predictions and excludes all records predicted to be irrelevant. To reduce the full text screening workload, the reviewer screens the records predicted to be relevant. Of these, those that the reviewer agrees are relevant move forward to full text screening.

2. In the context of dual independent screening (often used in systematic reviews):

- **Fully automated, dual independent screening approach**: after screening a training set of 200 records, the senior reviewer downloads the predictions. The second reviewer screens all of the records as per usual. Abstrackr’s predictions and the second reviewer’s decisions are compared and any marked as relevant by either the second reviewer, or the senior reviewer/Abstrackr move forward to full text screening; or
- **Semi-automated, dual independent screening approach**: after screening a training set of 200 records, the senior reviewer downloads the predictions and excludes all records predicted to be irrelevant. To reduce the full text screening workload, the senior reviewer screens the records predicted to be relevant. The second reviewer screens all the records as per usual. Abstrackr’s predictions and the second reviewer’s decisions are compared and any marked as relevant by either the second reviewer, or the senior reviewer/Abstrackr move forward to full text screening.

Appendix A includes a visual representation of each screening approach. To test the feasibility of the approaches, we downloaded Abstrackr’s relevance predictions for each review. In Excel (v. 2016, Microsoft Corporation, Redmond, Washington), we created a workbook for each review, including a row for each record and a column for each of: the title and abstract screening decisions (retrospective); the full text consensus decisions (retrospective); and Abstrackr’s relevance predictions. We then determined the title and abstract consensus decisions that would have resulted via each approach. Two researchers tabulated the results of each simulation, and compared their results to minimize the risk of error.

Comprehensive search strategies include not only searching bibliographic databases but scanning reference lists, searching trial registries and grey literature, and contacting experts [2, 14]. To determine whether the records missed by each approach would have been located via other means, one researcher (MG) performed a cited references search in Scopus and Google Scholar (for records not indexed in Scopus) to simulate scanning the reference lists of the included studies.

### Analysis

We exported all data to SPSS Statistics (v. 25, IBM Corporation, Armonk, New York) for analyses. Using data from 2 × 2 cross-tabulations, we calculated performance metrics for each approach using standard formulae: [4]

1. **Proportion of records missed** (i.e., error): of the records included in the final report, the proportion that were excluded during title and abstract screening.
2. **Workload savings** (i.e., absolute screening reduction): of the records that need to be screened by title and abstract, the proportion that would not need to be screened manually.
3. **Estimated time savings**: the time saved by not screening the records manually. We assumed a screening rate of 0.5 min per record [15] and an 8-h work day.

These performance metrics were selected because they (a) have been reported in previous published evaluations [8, 11], allowing for comparisons to other studies, and (b) are relevant to review teams and end users of reviews who are considering the balance of benefits and risks of adopting ML-assisted screening approaches. Appendix B shows the 2 × 2 tables and calculation of the performance metrics for one systematic review (Activity and pregnancy) and one rapid review (Community gardening).

## Results

### Screening characteristics and Abstrackr’s predictions

The predictions became available after the 200-record training set for all reviews, except Visual Acuity, for which we needed to screen 300 records (likely due to the small proportion of included studies). Table 2 shows the characteristics of the training sets and Abstrackr’s predictions for each review. The median (range) proportion of included records in the training sets was 7 (1 to 13)% for the systematic reviews and 25 (4 to 38)% for the rapid reviews. Abstrackr predicted that a respective median (range) 30 (12 to 67)% and 48 (10 to 65)% of the remaining records in the systematic and rapid reviews were relevant.

**Table 2:** Characteristics of the training sets and Abstrackr’s predictions for each review

Review name	Screening workload, n ^a^	Training set, n includes/excludes (% includes) ^b^	Predicted relevant by Abstrackr, n (%)
**Systematic reviews**			
**Biomarkers**	1812	14/186 (7)	503 (31)
**Brain injury**	6262	11/189 (6)	2126 (35)
**Activity and pregnancy**	2928	10/190 (5)	319 (12)
**Concussion**	1439	3/197 (2)	638 (51)
**Antipsychotics**	12,156	15/185 (8)	2117 (18)
**Digital technologies for pain**	2662	15/185 (8)	321 (13)
**Treatments for bronchiolitis**	5861	12/188 (6)	656 (12)
**VBAC**	5092	25/175 (13)	1490 (30)
**Visual acuity**	11,229	4/296 (1)	3639 (33)
**Experience of bronchiolitis**	651	13/187 (7)	111 (25)
**Experiences of UTIs**	1493	3/197 (2)	864 (67)
**Rapid reviews**			
**Preterm delivery**	451	47/153 (24)	95 (38)
**Community gardening**	1536	55/145 (28)	139 (10)
**Depression safety**	964	7/193 (4)	449 (59)
**Depression treatments**	1583	43/157 (22)	904 (65)
**Patient education for cancer**	2413	5/195 (3)	1410 (64)
**Workplace stress**	767	36/164 (18)	210 (37)

*UTI* urinary tract infection; *VBAC* vaginal birth after caesarean section

^a^Retrospective screening workload for each of the two reviewers in systematic reviews, and for the single reviewer in rapid reviews

^b^The training sets were 200 records for all reviews, with the exception of the Visual Acuity systematic review, for which 300 records were needed for Abstrackr to develop predictions

### Single reviewer simulations

Table 3 shows the performance metrics for the single reviewer approaches. For the fully automated approach, the median (range) proportion missed across the systematic reviews was 11 (0 to 38)%, or 7 (0 to 35) records in the final reports. The proportion missed for the semi-automated approach was 20 (0 to 44)%, or 9 (0 to 37) included records. Across the rapid reviews, the proportion missed was 6 (0 to 22)% for both the fully and semi-automated simulations, or 2 (0 to 25) included records. In all but two systematic reviews, the semi-automated and fully automated approaches resulted in more missed records than independent screening by a single reviewer (i.e., the second reviewer).

**Table 3:** Proportion missed, workload savings, and estimated time savings for the single reviewer simulations

Review name	Proportion missed, single reviewer, n (%) ^a^	Single reviewer, fully automated simulation			Single reviewer, semi-automated simulation		
		Proportion missed, n (%)	Workload savings, n (%)	Time savings, hours (days)	Proportion missed, n (%)	Workload savings, n (%)	Time savings, hours (days)
**Systematic reviews**							
**Biomarkers**	1 (2)	6 (13)	3424 (94)	29 (4)	20 (44)	2921 (85)	24 (3)
**Brain injury**	2 (5)	2 (5)	12,324 (98)	103 (13)	11 (28)	10,198 (81)	85 (11)
**Activity and pregnancy**	11 (11)	12 (12)	5656 (97)	47 (6)	17 (17)	5337 (91)	44 (6)
**Concussion**	0 (0)	0 (0)	2678 (93)	22 (3)	1 (20)	2040 (71)	17 (2)
**Antipsychotics**	4 (3)	35 (28)	24,112 (99)	201 (25)	37 (29)	21,995 (90)	183 (23)
**Digital technologies for pain**	0 (0)	7 (11)	5124 (96)	43 (5)	9 (14)	4803 (90)	40 (5)
**Treatments for bronchiolitis**	10 (7)	7 (5)	11,522 (98)	96 (12)	7 (5)	10,866 (93)	91 (11)
**VBAC**	5 (24)	8 (38)	9984 (98)	83 (10)	8 (38)	8494 (83)	71 (9)
**Visual acuity**	0 (0)	0 (0)	22,258 (99)	185 (23)	0 (0)	18,619 (83)	155 (19)
**Experience of bronchiolitis**	12 (43)	8 (29)	1102 (85)	9 (1)	9 (32)	991 (76)	8 (1)
**Experiences of UTIs**	0 (0)	0 (0)	2786 (93)	23 (3)	0 (0)	1940 (65)	16 (2)
**Rapid reviews**							
**Preterm delivery**	Not applicable	1 (3)	251 (56)	2 (< 1)	1 (3)	161 (36)	1 (< 1)
**Community gardening**	Not applicable	3 (9)	1336 (87)	11 (1)	3 (9)	1197 (78)	10 (1)
**Depression safety**	Not applicable	0 (0)	764 (79)	6 (< 1)	0 (0)	315 (41)	3 (< 1)
**Depression treatments**	Not applicable	25 (14)	1383 (87)	12 (1)	25 (14)	479 (30)	4 (< 1)
**Patient education for cancer**	Not applicable	0 (0)	2213 (92)	18 (2)	0 (0)	803 (33)	7 (< 1)
**Workplace stress**	Not applicable	13 (22)	567 (74)	5 (< 1)	13 (22)	357 (47)	3 (< 1)

*UTI* urinary tract infection; *VBAC* vaginal birth after caesarean section

^a^Proportion missed (retrospective) had the screening been completed by the second reviewer in isolation

For the fully automated approach, the median (range) workload savings across systematic reviews was 97 (85 to 99)%, or 5656 (1102 to 24,112) records that would not need to be screened manually. For the semi-automated simulation, the workload savings was 83 (65 to 93)%, or 5337 (991 to 21,995) records. Across the rapid reviews, the median (range) workload savings for the fully automated approach was 83 (56 to 92)%, or 1050 (251 to 2213) records. For the semi-automated approach, the workload savings was 39 (30 to 78)%, 418 (161 to 1197) records.

For the fully automated approach, the median (range) estimated time savings across systematic reviews was 47 (9 to 201) hours, or 6 (1 to 25) days. For the semi-automated approach, the time savings was 44 (8 to 183) hours, or 7 (1 to 23) days. For the rapid reviews, the time savings for the fully automated simulation was 9 (2 to 18) hours, or 1 (< 1 to 2) days. For the semi-automated simulation, the time savings was 3 (1 to 10) hours, or < 1 (< 1 to 1) day.

### Dual independent screening simulations

Table 4 shows the performance metrics for the dual independent screening approaches (relevant only to the systematic reviews). Across systematic reviews, the median (range) proportion missed was 0 (0 to 14)% for the fully automated approach, or 0 (0 to 3) records in the final reports. For the semi-automated simulation, the proportion missed was 1 (0 to 14)%, or 1 (0 to 6) included records. For six (55%) of the systematic reviews, fewer records were missed via the fully automated approach compared with independent screening by a single reviewer (i.e., the second reviewer). For the semi-automated simulation, the same was true for five (45%) of the systematic reviews.

**Table 4:** Proportion missed, workload savings, and estimated time savings for the dual independent screening simulations

Systematic review name	Proportion missed, single reviewer, n (%) ^**a**^	Dual independent screening, fully automated simulation			Dual independent screening, semi-automated simulation		
		Proportion missed, n (%)	Workload savings, n (%)	Time savings, hours (days)	Proportion missed, n (%)	Workload savings, n (%)	Time savings, hours (days)
**Biomarkers**	1 (2)	0 (0)	1612 (47)	13 (2)	6 (13)	1109 (32)	9 (1)
**Brain injury**	2 (5)	0 (0)	6062 (48)	51 (6)	0 (0)	3936 (31)	33 (4)
**Activity and pregnancy**	11 (11)	1 (1)	2728 (47)	23 (3)	1 (1)	2409 (41)	20 (3)
**Concussion**	0 (0)	0 (0)	1239 (43)	10 (1)	0 (0)	601 (21)	5 (< 1)
**Antipsychotics**	4 (3)	2 (2)	11,956 (49)	100 (12)	3 (2)	9839 (40)	82 (10)
**Digital technologies for pain**	0 (0)	0 (0)	2462 (46)	21 (3)	0 (0)	2141 (40)	18 (2)
**Treatments for bronchiolitis**	10 (7)	1 (1)	5661 (48)	47 (6)	1 (1)	5005 (43)	42 (5)
**VBAC**	5 (24)	3 (14)	4892 (48)	41 (5)	3 (14)	3402 (33)	28 (4)
**Visual acuity**	0 (0)	0 (0)	11,029 (49)	92 (11)	0 (0)	7390 (33)	62 (8)
**Experience of bronchiolitis**	12 (43)	1 (1)	451 (35)	4 (< 1)	1 (1)	340 (26)	3 (< 1)
**Experiences of UTIs**	0 (0)	0 (0)	1293 (43)	11 (1)	0 (0)	447 (15)	4 (< 1)

*UTI* urinary tract infection; *VBAC* vaginal birth after caesarean section

^a^Proportion missed (retrospective) had the screening been completed by the second reviewer in isolation

The median (range) workload savings was 47 (35 to 49)% for the fully automated simulation and 33 (15 to 43)% for the semi-automated simulation, accounting for a respective 2728 (451 to 11,956) and 2409 (340 to 9839) records that would not need to be screened manually. The median (range) estimated time savings was 23 (4 to 100) hours for the fully automated simulation and 20 (3 to 82) hours for the semi-automated simulation, equivalent to a respective 4 (< 1 to 12) and 3 (< 1 to 10) days.

### Cited references search

The dual independent screening, semi-automated approach provided the best balance of benefits and risks (i.e., relatively large workload savings and few missed records). We identified 10 (59%) of the 17 studies erroneously excluded across systematic reviews via the cited references search. This resulted in a reduction in the proportion missed among five (83%) of the six systematic reviews in which studies were missed. In the Biomarkers, VBAC, and Experiences of bronchiolitis reviews, the number of studies missed was reduced from 6 (13%) to 2 (4%), 3 (14%) to 2 (10%), and 3 (11%) to 1 (4%), respectively. In the Antipsychotics and Treatments for Bronchiolitis reviews, where a respective 3 (2%) and 1 (1%) studies were missed, all were successfully identified via the cited references search. Across systematic reviews, the median (range) proportion missed diminished to 0 (0 to 10)%, accounting for 0 (0 to 2) of the studies in the final reports.

## Discussion

We evaluated the risks and benefits of four approaches to leveraging a ML tool’s relevance predictions to expedite title and abstract screening in systematic and rapid reviews. Although the potential for workload and time savings were greatest in the single reviewer approaches, up to more than 40% of relevant studies were missed. We did not evaluate the impact of the missed studies on the reviews’ conclusions, but given the inherent risk it is unlikely that review teams would readily adopt the single reviewer approaches. Conversely, the dual independent screening approaches both resulted in few missed studies, and the potential time savings remained considerable, especially in reviews with larger search yields (e.g., up to an estimated 100 h in the Antipsychotics review). Balanced with the relatively small risk of missing relevant studies, the dual independent screening, semi-automated approach (which reduces the full text screening volume compared to the fully automated approach) may be trustworthy enough for review teams to implement in practice.

The gains in efficiency afforded by the automated and semi-automated approaches were less apparent among the rapid reviews compared with the systematic reviews. One means of expediting review processes in the rapid reviews was to limit their scope, and thus the search yield and number of records to screen. This, in addition to the fact that records were screened by a single reviewer, considerably limited the potential for gains in efficiency. Although limitations on scope and modifications to screening procedures are common [16] and well accepted [17] in rapid reviews, the potential for ML-assisted screening to expedite their completion should not be discounted. The slow adoption of ML has largely been influenced by review teams’ and end users’ distrust in a machine’s ability to perform at the level of a human reviewer [6]. Since end users of rapid reviews are sometimes more willing to compromise methodological rigour in order to obtain information to support decision-making sooner, rapid reviews may be an appealing medium for early adopters of ML-assisted screening.

Our findings are supportive of methods whereby a ML tool’s predictions are used to complement the work of a human reviewer. Although the proposed approaches are admittedly less trustworthy (albeit slightly) than dual independent screening, to fully appreciate their potential, the findings must be interpreted in context. In rigorously conducted systematic reviews, electronic database searches are supplemented with additional search methods, e.g., contacting experts, hand-searching grey literature, so the limited risk of missing relevant records would be further diminished. As we have demonstrated, most missed records are likely to be identified via reference list scanning alone. We also speculate that any large, well-conducted study that would change the findings of a review would be identified by conscientious review teams at some point during the evidence synthesis process.

### Strengths and limitations

Building on earlier studies that evaluated Abstrackr [8–11], we used a heterogeneous sample of reviews to compare and contrast the benefits and risks for four approaches to leveraging its relevance predictions. We used cited references searches to determine if missed studies would have been located via other means, simulating real-world evidence synthesis methodology. Although human reviewer judgement is imperfect, in this study it provided a realistic reference standard against which to compare the automated and semi-automated screening approaches.

Although the training set was sufficient, in most cases, to bring about predictions, it is possible that another training set size would have resulted in different findings. Research at our centre showed that modest increases in the training set size (i.e., 500 records) did not improve upon the reliability of the predictions [11]. Whether the missed studies would affect the conclusions of reviews is an important concern for review teams; however, we did not evaluate this outcome. So few studies were missed via the dual independent screening approaches that substantial changes to review findings are highly unlikely.

The retrospective nature of this study did not allow for precise estimates of time savings. Potential gains in efficiency were estimated from a standard screening rate of two records per minute, as reported in an earlier study [15]. Although the selected screening rate was ambitious, it provided for conservative estimates of time savings for the purpose of this study.

## Conclusions

Using Abstrackr’s relevance predictions to assist one of two reviewers in a pair saves time while posing only a small risk of missing relevant studies in systematic reviews. In many cases, the approach was advantageous compared with screening by a single reviewer (i.e., fewer studies were missed). It is likely that missed studies would be identified via other means in the context of a comprehensive search. In the circumstance of screening via a single reviewer (i.e., in rapid reviews), the time savings of the fully and semi-automated approaches were considerable; however, adoption is unlikely due to the larger risk of missing relevant records.

## Supplementary information

**Additional file 1.** Retrospective Screening Simulations

**Additional file 2 Appendix A.** 2 × 2 tables and performance metrics calculation for a systematic review and a rapid review.

## Data Availability

The datasets generated and analyzed during the current study are available from the corresponding author on reasonable request.

## Abbreviations

ML: Machine learning
VBAC: Vaginal birth after caesarean section
