# Supplementary material for: The semi-automation of title and abstract screening: a retrospective exploration of ways to leverage Abstrackr’s relevance predictions in systematic and rapid reviews
Source: BMC Med Res Methodol. 2020 Jun 3;20:139. doi: 10.1186/s12874-020-01031-w (PMC7268596; doi:10.1186/s12874-020-01031-w)

**Supplementary File 1.** Retrospective Screening Simulations

**1a.** Fully automated, single screener approach

**
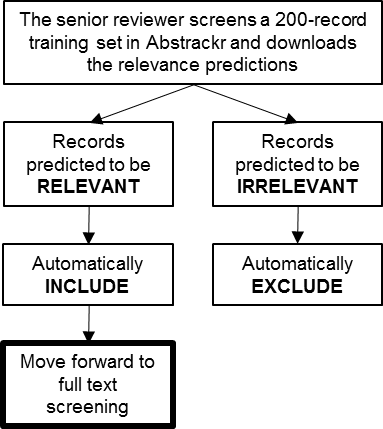
**

**1b.** Semi-automated, single screener approach

**
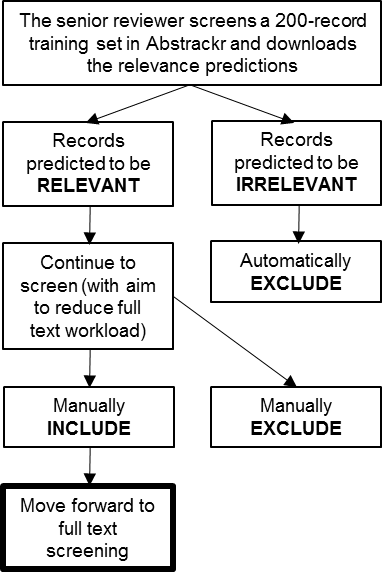
**

**2a.** Fully automated, dual independent screening approach

**
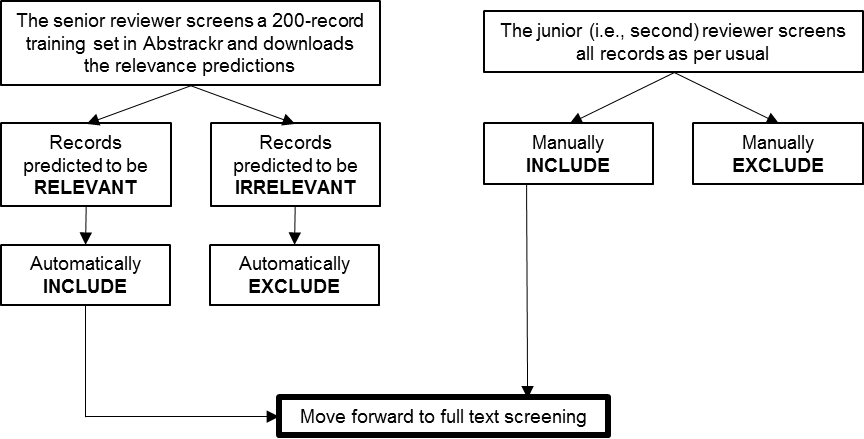
**

**2b.** Semi-automated, dual independent screening approach


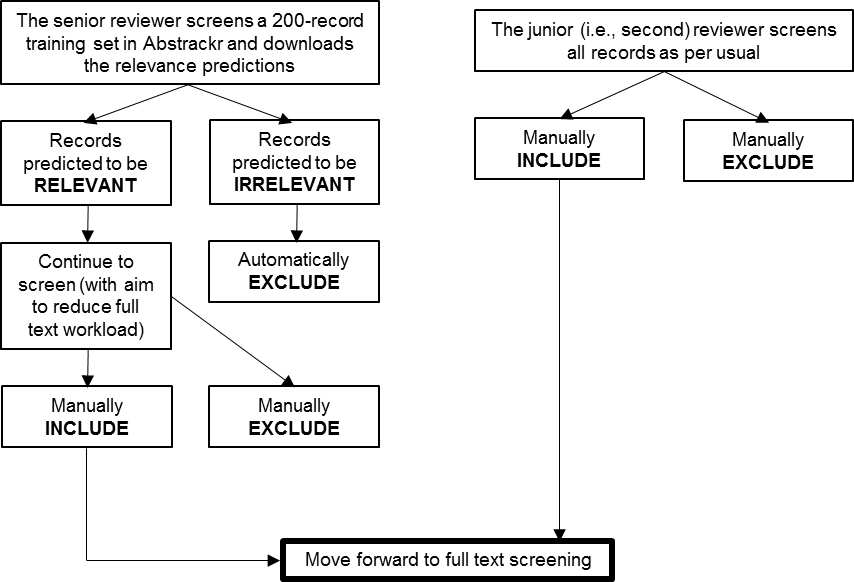

Supplement: Supplementary file 1 — Additional file 1. Retrospective Screening Simulations [file 12874_2020_1031_MOESM1_ESM.docx]
