# Supplementary material for: The semi-automation of title and abstract screening: a retrospective exploration of ways to leverage Abstrackr’s relevance predictions in systematic and rapid reviews
Source: BMC Med Res Methodol. 2020 Jun 3;20:139. doi: 10.1186/s12874-020-01031-w (PMC7268596; doi:10.1186/s12874-020-01031-w)
Supplement: Supplementary file 2 — Additional file 2 Appendix A. 2 × 2 tables and performance metrics calculation for a systematic review and a rapid review. [file 12874_2020_1031_MOESM2_ESM.docx]

**Appendix A.** 2x2 tables and performance metrics calculation for a systematic review and a rapid review

**Systematic Review:** Activity and pregnancy

After screening 200 records, Abstrackr predicted that 2409 of the remaining records were irrelevant.

**Table A.1. Single reviewer automated simulation**

|  | Excluded from final report | Included in final report | Row total |
| --- | --- | --- | --- |
| Excluded by Simulation | 2587 | 12 | 2599 |
| Included by Simulation | 243 | 86 | 329 |
| Column total | 2830 | 98 | 2928 |

Proportion missed = 12/98 = 0.12 or 12%

Workload savings = [(2928 x 2) – 200] / (2928 x 2) = 0.97 or 97%

Time savings = ([2928 x 2) – 200] records x 0.5 minutes/record x 1 hour/60 minutes = 47 hours = 6 days

**Table A.2. Single reviewer semi-automated simulation**

|  | Excluded from final report | Included in final report | Row total |
| --- | --- | --- | --- |
| Excluded by Simulation | 2784 | 17 | 2801 |
| Included by Simulation | 46 | 81 | 127 |
| Column total | 2830 | 98 | 2928 |

Proportion missed = 17/98 = 0.17 or 17%

Workload savings = (2928 + 2409) / (2 x 2928) = 0.91 or 91%

Time savings = (2928 + 2409) records x 0.5 minutes/record x 1 hour/60 minutes = 44 hours = 6 days

**Table A.3. Dual independent screening automated simulation**

|  | Excluded from final report | Included in final report | Row total |
| --- | --- | --- | --- |
| Excluded by Simulation | 2527 | 1 | 2528 |
| Included by Simulation | 303 | 97 | 221 |
| Column total | 2830 | 98 | 2928 |

Proportion missed = 1/98 = 0.01 or 1%

Workload savings = (2928 – 200) / (2 x 2928) = 0.47 or 47%

Time savings = (2928 – 200) records x 0.5 minutes/record x 1 hour/60 minutes = 23 hours = 3 days

**Table A.4. Dual independent screening semi-automated simulation**

|  | Excluded from final report | Included in final report | Row total |
| --- | --- | --- | --- |
| Excluded by Simulation | 2706 | 1 | 2707 |
| Included by Simulation | 124 | 97 | 221 |
| Column total | 2830 | 98 | 2928 |

Proportion missed = 1/98 = 0.01 or 1%

Workload savings = 2409 / (2 x 2928) = 0.41 or 41%

Time savings = 2409 records x 0.5 minutes/record x 1 hour/60 minutes = 20 hours = 3 days

**Rapid review:** Community gardening

After screening 200 records, Abstrackr predicted that 1197 of the remaining records were irrelevant

**Table A.5. Single reviewer automated simulation**

|  | Excluded from final report | Included in final report | Row total |
| --- | --- | --- | --- |
| Excluded by Simulation | 1339 | 3 | 1342 |
| Included by Simulation | 165 | 29 | 194 |
| Column total | 1504 | 32 | 1536 |

Proportion missed = 3/32 = 0.09 or 9%

Workload savings = (1536 – 200) / 1536 = 0.87 or 87%

Time savings = (1536 – 200) records x 0.5 minutes/record x 1 hour/60 minutes = 11 hours = 1 day

**Table A.6. Single reviewer semi-automated simulation**

|  | Excluded from final report | Included in final report | Row total |
| --- | --- | --- | --- |
| Excluded by Simulation | 1418 | 3 | 1421 |
| Included by Simulation | 86 | 29 | 115 |
| Column total | 1504 | 32 | 1536 |

Proportion missed = 3/32 = 0.09 or 9%

Workload savings 1197/1536 = 0.78 or 78%

Time savings = 1197 records x 0.5 minutes/record x 1 hour/60 minutes = 10 hours = 1 day
